# Why bother: usefulness and effect of young surgeon committees in surgical societies

**DOI:** 10.1515/iss-2018-0034

**Published:** 2018-12-13

**Authors:** Brigitta M. Lutz, Benedikt J. Braun, Beate Blank, Manuel Mutschler

**Affiliations:** Department for Visceral, Thoracic and Vascular Surgery, University Hospital Carl Gustav Carus Dresden, Dresden, Germany; Department of Trauma, Hand and Reconstructive Surgery, Saarland University Hospital, Homburg, Germany; Plastische und Handchirurgie, Klinikum Kulmbach, Kulmbach, Germany; Department of Traumatology and Orthopedic Surgery, Witten/Herdecke University, Cologne-Merheim Medical Center (CMMC), Koeln, Germany

**Keywords:** societal participation, surgeons in training, surgical society, surgical training, young surgeons board

## Abstract

Work-time constraints during surgical residency along with managing a private life usually take up the majority of the time of young surgeons. For many, work with a surgical society seems like something neither generally promising nor personally worthwhile, thus raising the question, why bother? This article sets out to show examples of the effects that surgical societies and young surgeon committees can have on surgery and residency training. Additionally, we highlight the personal side of being active on a committee. Our aim is to raise interest in participating in societal work by showing the rewarding general effects as well as personal benefits. While this article is based primarily on experiences made in Germany, we believe that aspects can be transferred to other medical systems.

## Introduction

Usually, work as a trainee in a surgical unit consumes the majority of the time of surgical residents. Between managing the acquisition of technical skills and required knowledge, the commitment in research or teaching, and private life, the remaining time is sparse. More surgical residents start raising children during their training [1], and the time for and interest in research is dropping [2], while workload and relative discontent with the working situation remains high [3]. Further, the active members of surgical societies are frequently university professors or other heads of surgical departments, hence raising the question, why should a trainee bother to be part of a surgical society?

When starting out as a young surgical resident, many changes come to the life we used to know as students, bringing up new challenges, issues, and questions. Naturally, the main goal of a surgical resident is to be well trained and educated in a manageable period of time. Yet, at the same time, most would agree that there should also be an equal part for the other things in life, such as family, hobbies, teaching, and research, just to name a few. Even without societal work, this equals a full schedule for most surgical residents. The challenge is to become a skilled surgeon and a scientist while getting one’s exercise, meeting friends, traveling, and having an active family life. This is commonly referred to as work-life balance or blending – highly individual, yet concerning everyone [4].

One of the most pressing concerns during residency training is starting a family. Depending on the subspecialty chosen, >60% of residents start a family during their training [1], and this is a major issue even for younger medical students [3]. Due to societal and demographic changes, most parents like to take an increasingly larger and active part in raising their children. As a result, both male and female surgeons take some time off the job to care for their kids. This break seems to compete with quick and efficient training in surgery. Additionally, in Germany, the majority of medical school graduates are women at present, resulting in an increase in the number of female surgeons as well. Female surgeons might take several breaks during pregnancy and parenting, or return to the job as part-time employees. Solutions exist locally [5], but on the national level, this remains a challenge.

These circumstances together with advancing surgical techniques result in changing conditions in surgery itself and, therefore, also in surgical training. We have to think outside the box in order to bring together the needs and advantages of both the surgical field and the trainees. Surgical training has to become more flexible and at the same time cover extensive knowledge and skills.

To positively contribute to these changes on a general level and on the societal level is one of the main issues for young surgical committees. It concerns all of us and should therefore be of interest to the majority of surgeons. Yet, because of many reasons, participation in these committees is depressingly low. The aim of this article is to show some of the effects of such work on both the general and personal levels, to potentially encourage the future generation of surgeons to actively participate in shaping our surgical future. Although this article is based primarily on experiences made in Germany, we believe that aspects can be transferred to other medical systems.

## Tasks of surgical societies and their young professional committees

### Promote surgery to young professionals

One of the major tasks of surgical societies is to ensure the future of the surgical profession through the acquisition of fitting young professionals in a sufficiently large number. Shortages of well-trained surgeons will eventually also diminish the impact of surgery among the other medical fields. Surgical disciplines have to deal with a decreasing number of candidates for surgical training [6]. On the one hand, the times when applications piled up high on the desks of professors and heads of departments are very much over. On the other hand, this offers a seemingly comfortable situation in the job market for newcomers. The question that is raised nowadays is not “will I find a job?” anymore, but rather “where do I like to work?” or “how do I like to spend my working hours?” – questions to which surgery does not always have the best answers. Furthermore it seems like we are shying students away the more they are exposed to our daily surgical life in internships. Soft influencing factors through positive role models and mentoring in surgery are among the most important elements to motivate students to follow surgical careers, and surgical residents to subspecialize [7], [8]. It seems like these actions are ones that can only be taken on a local or personal level. Yet, there are many problems that should and can be addressed by surgical societies. Surgical societies offer plenty of room for personal interactions not only through their annual meetings but also through local events that are targeted to both students and young professionals. Special programs for students in surgical expert conferences and several recruiting and teaching campaigns of the Professional Association of German Surgeons [9], [10] are excellent examples of events that directly influence and positively motivate students to pursue a surgical career (see Table 1). This direct contact with young, interested students is one of the key motivators to not only pursue a surgical career but also to become involved in young surgeon committees.

**Table 1:** Support programs in Germany for students or young surgeons.

Kongress der Deutschen Gesellschaft für Chirurgie
This is the annual meeting of the German Surgical Society. With over 4000 visitors, it represents research from all aspects and subspecialties of surgery. Each year, >50 students are invited to visit the meeting for free, with travel and lodging costs provided by the surgical societies and sponsors. A special program addressed to students consisting of informational talks and a skills laboratory is provided.
Nur Mut: ChirurgIn werden
“Nur Mut” is a campaign of the Professional Association of German Surgeons aimed at introducing students to surgery. They offer practical courses and meetings all over Germany, provide help with job applications, and run a successful website providing free additional information on topics such as career and family.
Staatsexamen und Karriere
This is a student course jointly run by the Professional Association of German Surgeons and the German Society of Internal Medicine. They provide comprehensive repetition courses and practical courses for students preparing for their final medical examinations.
Operieren in der Schwangerschaft (OPIDS)
OPIDS is an initiative of young female surgeons to check which kinds of surgery could be performed by pregnant women. OPIDS offers information on practical and legal questions for those who like to continue working in the operation theater while pregnant.

Apart from the personal level, the major points of complaints among surgical trainees should become the key focus areas of surgical societies to increase the attractiveness of surgery. Among these complaints is the lack of structured training programs and, with that, the absence of a projectable career. Furthermore, with the rising desire for a balanced work and private life, surgery faces a natural loss in popularity owing to the expected extensive working hours [11]. The quality and attractiveness of surgical training can be enhanced by a structured curriculum, a frequent dialogue between trainee and supervisor, a sufficient and comparable log book, indication meetings, planned rotations, internal and external training options, feedback, train-the-trainer concepts, and mentoring [11]. Surgical societies can create and submit template curricula together with recommendations for rotations and external trainings. Also, they are involved in forming recommendations for specific subspecialty trainings, which are otherwise left to the federal chambers of physicians. Through internal surveys and studies, they can benchmark external training offers and organize training units in a structured fashion. Training recommendations and programs can then be customized to the local curriculum, so that it matches the local conditions. Surgical societies can help supervisors achieve these goals with guidelines and train-the-trainer workshops.

The German authorities have established a new training program for all medical fields. This is an important chance to shape the standards so that they fit the needs and expectations of both the trainees and the supervisors. It is crucial to again increase the attraction of surgery as a medical profession, especially among those whose assets and interests would suit surgery very well. Otherwise, we will lose these gifted and motivated young professionals either to other medical disciplines or even to areas outside of medicine. As young surgeons, we are in the same generation as the potential new candidates for the field of surgery. We share their needs and worries. Therefore, we are the perfect partners to identify and erase potential pitfalls and obstacles toward a surgical career.

### Support female surgeons in their professional advancement

Another factor that reduces applicants for surgery is the gender aspect. Many potential female candidates are unsure whether surgery is compatible with an active family life. Those medical students who have a strong wish for a family life are less likely to choose a career in surgery [12]. A surgical society should create a framework and offer support in order to make surgical training possible for those women who consider both a career in surgery and having a family. In Germany, many pregnant surgeons announce their pregnancy rather late, as a consequence of very restrictive laws. Some of the restrictions and regulations are very reasonable and important, like the prohibition of working at night and providing adequate time for breaks. It is known that the number of obstetrical complications rises with the number of nights on call and with longer operating hours [13]. However, these laws also prohibit the majority of daily surgical tasks. Pregnant women are usually only allowed to cover administrative tasks, and thus have no training in manual surgical skills. Together with the maternity leave, which is usually taken after birth, this even widens the gap between female and male surgeons in their professional advancement. Even though pregnancy is a special condition, women should not be excluded entirely from the operating theater. Usually, every surgical discipline offers procedures that are short and could be done without volatile anesthetics or the use of fluoroscopy. On the one hand, it is important that pregnant women are protected especially when it comes to working conditions. On the other hand, they should have the chance to continue their surgical training. One major effort to address this topic is OPIDS (see Table 1), a program that offers structured advice on how to manage an active surgical career while being pregnant and can certainly be considered as a great success for surgery all over Germany [14]. This project has been launched by a young surgeon committee.

Aside from pregnancy and parenting, female surgeons sense a certain lack of gender equality in their working environment [15]. While the number of female surgical residents constantly increases, there is still a low number of females in academic surgical departments [16] and a very low number of female full professors in surgery [17]. With surgery becoming more feminine in many countries, surgical societies do not need to encourage their members to employ more female candidates. This is already done on a regular basis. Surgical societies should rather create programs to support female surgeons during their career, with special attention to leading positions and research commitments. Special mentoring programs, especially same-sex mentorship [18], could be very supportive in this context.

### Distinction from and cooperation with other fields

Technological progress permits new possibilities in both surgery and in other medical fields. It is the responsibility of surgical societies to share knowledge of new technologies and to train their members in these new skills. Otherwise, we will not remain capable of competing with other medical fields such as internal medicine or interventional radiology. At the same time, we need to stay in close dialogue with other medical societies, especially for patients who require knowledge and skills from several different medical fields. Eventually, the patient is our customer and every health-care participant has to treat this customer to the best of one’s knowledge and belief. As young surgeons, we are particularly interested in learning and mastering new procedures because the surgeon of the future might not use the same skills as the surgeon of today. Furthermore, our generation is interested in working together in harmonious cooperation, a perfect precondition for our interdisciplinary work life.

### Have political impact

Surgical societies need to represent their interests also toward political leaders, the medical self-administration, insurance companies, and hospital management. As professionals in the field, they have to provide insights into surgical knowledge to decision makers. Especially when treatment options are new or not well evaluated yet, surgical societies need to offer a reliable assessment, so that payment providers can choose whether treatment costs should be covered and legislators would know whether a certain treatment option is safe for the patients. Political leaders wish to establish a minimum number of cases for the treatment of a certain diagnosis. Surgical societies have to check whether these numbers of cases are achievable and whether they allow nationwide health-care coverage. We cannot watch politicians change the situation just to suit their agenda or populist goals. We have to be a strong partner who claims the profession’s interests and those of the patients.

As members of a young surgical committee, our influence is through position papers and statements in local and national media. Furthermore, personal influence can be built through advisory functions on local and national political boards. Yet, with few members, our voices can easily be ignored. With more members, both surgical societies in general and young surgeon committees in particular can certainly acquire more influence. With more influence, we might take a bigger part in creating our own future. A young surgeon should participate in the renewal of surgical training programs and in promotional campaigns for students. Young surgical committees can offer all this while also providing a network platform that can remain throughout a professional career.

## The benefits in brief

– Active participation in increasing the attractiveness of surgery, through
  – regulatory work on committees or
  – guidelines and position papers concerning work and surrounding conditions.
    – Placing special focus on women in surgery.
    – Digitalization.
    – Workspace of the future.
– Working with students and increasing their motivation toward a career in surgery, through
  – local and national workshops,
  – student meetings and preparatory courses, and
  – mentoring programs.
– Networking with other surgeons and physicians overall to
  – increase the reach of projects and
  – foster collaborations.

## The personal side

In addition to the overall benefits of societal work, the aim of this article is to introduce personal statements to the reader. We would like to present why some of our members chose to work for a surgical society in addition to having a full work schedule.

### Beate Blank

Many of my colleagues have yet to understand the reasons for me investing my personal time into the membership of a young surgeon board. For me, it means trying to change some basic conditions and behaviors that are so deeply rooted in surgery itself, people do not even question them anymore.

I got involved simply because I love surgery. I wanted to become a doctor since I was 3 years old and wrote out prescriptions to my kindergarten classmates. As I grew older and learned more about the human body, I got fascinated with the human body rather than being disgusted by intestines and body fluids, and when I ultimately learned what surgeons do, it turned me into a 15-year-old with the same ambitions that I have today.

Why being involved is actually important to me is a different story. I met amazing young women in my first year of medical school who were thriving for the same goal as I was; however, as the years went by and we learned about the true meaning of becoming a surgeon, when it was time to graduate I was the only one left who did not want to let go of that dream. Gender aside, in my final year, we were only a handful of people in my class who were still interested in pursuing a career in surgery. For me, there has never been an alternative to surgery, and until today it frustrates me to see that there are people who give up surgery despite their interests, even passions, simply because of general conditions and negative experiences.

I have heard every reason not to become a surgeon, of course also because I am a young woman. With their rules of behavior and their expectations toward themselves, our predecessors have built up walls to accentuate themselves from other doctors. This has long become obsolete in today’s context and need to be broken down if surgery does not want to seem so repulsive to medical students. Smaller aspects including lunch breaks, working hours, and intercollegial communication but also more complicated matters like fair and structured teaching plans, employment law, maternity leaves and childcare, or the general perception of surgery can be changed. They will have to be changed, in order for surgery to survive and be an attractive career for young doctors, by people who will be around to witness the aftereffects.

I am involved for the same reason I am a surgeon. I am passionate about seeing the changes and improvements of my own work.

### Benedikt Braun

What made me get involved with a young surgeon committee? Honestly, chance and a perceived opportunity to improve my surgical career. Not the noblest of reasons, but the truth. However, that is not what got me to stay. As a student and also a young resident, I never quite understood what it meant to be involved in a surgical society and specifically in a young surgeon committee. You mostly saw the same faces at the conferences and figured they must be careerists for their own good. Once you have worked with these committees, however, the whole perspective changes. All of the surgeons involved I have gotten to meet to this point are out to improve the little and the large things that can be changed about our work situation. They invest large amounts of their free time and energy to change the way we all work for the better. For me, personally, it is on the one hand the chance to directly interact with interested students and young surgeons from all over Germany through workshops and special initiatives for students and represent the true spirit of surgery apart from the surrounding conditions that are limiting our profession today. On the other hand, it is the opportunity to actually change the conditions of our work environment through raising awareness with surveys and position statements, as well as directly influencing the future course of our residency training. Lastly, it is the interaction with the people involved, all passionate about surgery and easy to be around with. These people make it worthwhile to invest free time and energy.

During my time working on a young surgeon committee, we have already made it possible for students to participate for free in the German Surgical Society. We implemented changes to student courses, increasing their attractiveness and effect on young surgeons and are in the process of combining the efforts of different young surgeon committees, developing new study material for students and an online mentoring program.

### Brigitta Lutz

When I did my practical year, I almost lost track of surgery. There were some surgical professors who humiliated their employees. Maybe they had the impression that this would make them work harder. For us as students, it had the reverse effect. No one ever thought of applying in those departments. Fortunately, I also met some very great surgeons who served as good role models. They gave me positive feedback and the impression that a nice working environment in surgery does exist. That made me stay with the decision to become a surgeon. So when I started working, I decided to pass on this positive feedback to the students I taught. Then, a friend recommended me to become part of a young surgeon committee. The idea of a fruitful dialogue and interaction with other surgeons of my age excited me from the beginning. Our meetings served as a good forum to discuss many different subjects, from daily obstacles to a general preparation of one’s career. For 4 years, I supported the young surgeon board of the German Society of Vascular Surgery and Medicine as a representative speaker. I had the opportunity to take part in shaping sessions of scientific meetings and to discuss the role of young surgeons in our community. We introduced the participation of one young surgeon as third chair in every scientific session as an integral part of the annual scientific meeting. Last but not least, I got to know a couple of really nice people I am always happy to meet again.

### Manuel Mutschler

I became involved in a young surgeon committee of our society for orthopedics and traumatology (Junges Forum O&U) during university, namely by two young and motivated doctors who inspired me to be part of this community. After finishing medical school, I got quickly involved in the organization of different programs trying to motivate other students to become a trauma and orthopedic surgeon. We were able to present the whole variety of our profession, theoretically and practically.

During my own training, I recognized that there are several demanding topics coming up with our generation, which needed to be addressed. A good medical education in times of increasing work-time restrictions as well as the wish of combining a surgical career and family make new concepts and ideas necessary, which can only be developed by the young generation. Luckily, our society is open-minded and asks actively for the opinion of young, upcoming surgeons. Thus, we were able to organize workshops on topics like how to combine family and career in the future as well as how your training should be organized in the future. Taken together, on the one hand, one has to invest a lot of time and energy, but on the other hand, it is inspiring and worthwhile being part of a group of young surgeons who want to improve training and working conditions for the next generation of residents and fellows. Last, one has the chance also to be part of the steering committee of our society, which allows a deep insight and personal contacts to leading representatives of our society.

## Limitations

Obviously, an opinion article such as this has limitations, especially concerning the available literature. Some of the effects of the programs mentioned have not been extensively studied, and their outcome evaluation is at times based on brief surveys and personal experience. However, we feel that they are worth reporting, as change in certain aspects is needed and might come too slowly if only level 1 evidence is used to trigger change. Furthermore, the situations and institutions described in this article are focused on Germany, as this is the home medical system for all authors and the young surgeon committees involved in this project. Differences between systems will certainly limit the transferability of the results. Nonetheless, we feel that this article and the concepts and motivations can serve as an example for other medical systems.

## Conclusion

If you think about surgery just as a job to cover your personal expenses, you might not be interested in societal work and the chances are high that you have not been reading to this point. There is no denying that work on a surgical society is time consuming. It is unpaid, done mostly during free time, and requires personal effort that goes beyond the occasional meeting. However, from a general as well as personal point of view, the benefits are undeniable.

We hope that this article has raised your interest in contributing to societal work and especially young surgical committees, through active participation or just by visiting one of our many sessions at the next annual meeting. If you are a senior surgeon, spread the word, lead by example, and get your residents interested in actively changing our surgical community.

We are looking forward to seeing many new faces at one of our next sessions.

## Supplementary Material

The article (iss-2018-0034) offers reviewer assessments as supplementary material.
