# Supplementary material for: Why bother: usefulness and effect of young surgeon committees in surgical societies
Source: Innov Surg Sci. 2018 Dec 13;4(1):35–41. doi: 10.1515/iss-2018-0034 (PMC6754062; doi:10.1515/iss-2018-0034)
Supplement: Supplementary file 1 [file iss-4-20180034-s001.pdf]

Reviewer Assessment

Brigitta M. Lutz*, Benedikt J. Braun, Beate Blank and Manuel Mutschler

Why bother: usefulness and effect of young surgeon committees in surgical societies

https://doi.org/10.1515/iss-2018-0034
Received October 1, 2018; accepted November 28, 2018

***Corresponding author: Brigitta M. Lutz,** Department for Visceral, Thoracic and Vascular Surgery, University Hospital Carl Gustav Carus Dresden, Dresden, Germany, E-mail: Brigitta.Lutz@uniklinikum-dresden.de

Reviewers' Comments to Original Submission

Reviewer 1: anonymous

Oct 28, 2018

**Reviewer Recommendation Term:**	Revise with Major Modifications
**Overall Reviewer Manuscript Rating:**	30
**Custom Review Questions**	**Response**
Is the subject area appropriate for you?	5 - High/Yes
Does the title clearly reflect the paper's content?	4
Does the abstract clearly reflect the paper's content?	3
Do the keywords clearly reflect the paper's content?	4
Does the introduction present the problem clearly?	4
Are the results/conclusions justified?	4
How comprehensive and up-to-date is the subject matter presented?	3
How adequate is the data presentation?	N/A
Are units and terminology used correctly?	N/A
Is the number of cases adequate?	N/A
Are the experimental methods/clinical studies adequate?	N/A
Is the length appropriate in relation to the content?	4
Does the reader get new insights from the article?	1 - Low/No
Please rate the practical significance.	2
Please rate the accuracy of methods.	N/A
Please rate the statistical evaluation and quality control.	N/A
Please rate the appropriateness of the figures and tables.	N/A
Please rate the appropriateness of the references.	4
Please evaluate the writing style and use of language.	1 - Low/No
Please judge the overall scientific quality of the manuscript.	2
Are you willing to review the revision of this manuscript?	Yes

Open Access. © 2018 Lutz B.M. et al., published by De Gruyter. BY-NC-ND This work is licensed under the Creative Commons Attribution-NonCommercial-NoDerivatives 4.0 License.

Comments to Authors:

This manuscript deals with important problems of German Surgeons. Nevertheless, the are major concerns. First, the author want to publish in an international journal and their paper might be read by surgeons/doctors of different countries. The situation described and problems adressed in this paper are specific for Germany. This issue needs to be clarified.
Second, the English is not adequate (multiple syntax/grammar mistakes, unusual verbalisations) and needs to be revised by a native speaker. For example:
Student days at congresses such as the “Deutsche Chirurgen Kongress” but also events like “Nur Mut - ChirurgIn werden” [9] and “Staatsexamen und Karriere” [10] by the german berufsverband are excellent examples
> An international readership need translations and adequate explanations regarding the institutions
If surgery is simply a means to provide food and money for you and your family this article and work on a society is not for you and you‘ll probably not get to this point in reading anyways.
> I get the point but the sentence is difficult to understand and consists of multiple linguistic mistakes

Reviewer 2: anonymous

Oct 31, 2018

**Reviewer Recommendation Term:**	Revise with Major Modifications
**Overall Reviewer Manuscript Rating:**	33

**Custom Review Questions**	**Response**
Is the subject area appropriate for you?	4
Does the title clearly reflect the paper’s content?	5 - High/Yes
Does the abstract clearly reflect the paper’s content?	3
Do the keywords clearly reflect the paper’s content?	4
Does the introduction present the problem clearly?	2
Are the results/conclusions justified?	2
How comprehensive and up-to-date is the subject matter presented?	2
How adequate is the data presentation?	N/A
Are units and terminology used correctly?	N/A
Is the number of cases adequate?	N/A
Are the experimental methods/clinical studies adequate?	N/A
Is the length appropriate in relation to the content?	3
Does the reader get new insights from the article?	2
Please rate the practical significance.	1 - Low/No
Please rate the accuracy of methods.	N/A
Please rate the statistical evaluation and quality control.	N/A
Please rate the appropriateness of the figures and tables.	N/A
Please rate the appropriateness of the references.	2
Please evaluate the writing style and use of language.	1 - Low/No
Please judge the overall scientific quality of the manuscript.	1 - Low/No
Are you willing to review the revision of this manuscript?	Yes

Comments to Authors:
Overall the article raises an important topic on surgical education and the surgical future and should be considered for publication. Nevertheless, major revisions should be performed. You state following in your abstract:
“This article sets out to show the effects, that surgical societies and young surgeon committees can have on surgery and residency training, whilst also highlighting the personal side of being active on a committee. Our aim is to raise interest for participating in societal work by showing the rewarding general effects, as well as personal benefits.” This is achieved only partially after reading the article. The structure of the article can be improved regarding the aim of the paper.
Very importantly, German institutions have to be explained to the international readership:
For example: “events like “Nur Mut - ChirurgIn werden” [9] and “Staatsexamen und Karriere” [10] by the german berufsverband”. A non German reader will not understand what that means and what kind of event those are.
You talk about lot of gender issues in surgery, the discussion of the literature as well as the discussion of the current guidelines for pregnant surgeons could be more thorough. What about “#meetoo in medicine/surgery? There is a lot of international literature available on adverse events in pregnancy for surgical residents as well as publications on gender specific quality of treatement for female vs male surgeons. How can these problems be affected with more active societal work, expecially what can young surgeons do? You mentioned some, but overall the discussion should be more scientific.

I would rephrase the section “The Personal side” in “Personal motivation”
Regarding the section of personal motivation:
one should consider if the names should be mentioned. My suggestion would be:
Male/female, age, training status (intern/resident/with training year). I would also wish to hear more about what the authors/persons mentioned have already achieved for them or others through the work in a surgical society.

Authors’ Response to Reviewer Comments

Nov 19, 2018

Reviewer #1:

1) This manuscript deals with important problems of German Surgeons. Nevertheless, the are major concerns. First, the author want to publish in an international journal and their paper might be read by surgeons/doctors of different countries. The situation described and problems adressed in this paper are specific for Germany. This issue needs to be clarified.

Answer: Thank you for this important comment. We agree that this journal is an international journal and aimed at a diverse readership. Yet it is also the official journal of the German Surgical Society and the Special Issue for which the article is submitted is that of the Young Surgeons Forum, of that very society, so the primary readership might be of German origin. Regardless, we have now addressed this shortcoming on numerous occasions throughout the manuscript and have discussed this limitation. Also we provided a table explaining some of the specific institutions or initiatives in Germany referred to in the manuscript.

2) Second, the English is not adequate (multiple syntax/grammar mistakes, unusual verbalisations) and needs to be revised by a native speaker. For example: Student days at congresses such as the “Deutsche Chirurgen Kongress” but also events like “Nur Mut - ChirurgIn werden” [9] and “Staatsexamen und Karriere” [10] by the german berufsverband are excellent examples

> An international readership need translations and adequate explanations regarding the institutions

Answer: Extensive revisions of the written English were performed. A table explaining the above mentioned institutions is now provided.

3) If surgery is simply a means to provide food and money for you and your family this article and work on a society is not for you and you‘ll probably not get to this point in reading anyways.

> I get the point but the sentence is difficult to understand and consists of multiple linguistic mistakes

Answer: This sentence has been revised.

Reviewer #2:

1) Overall the article raises an important topic on surgical education and the surgical future and should be considered for publication.

Answer: Thank you.

2) Nevertheless, major revisions should be performed.

You state following in your abstract: “This article sets out to show the effects, that surgical societies and young surgeon committees can have on surgery and residency training, whilst also highlighting the personal side of being active on a committee. Our aim is to raise interest for participating in societal work by showing the rewarding general effects, as well as personal benefits.” This is achieved only partially after reading the article. The structure of the article can be improved regarding the aim of the paper.

Answer: Thank you for the valid comment. Literature on this comment is scarce and a truly evidence based approach to this topic cannot be provided. As such, an opinion paper such as ours will always be limited. We added a little more “soft” evidence. We have reworked the manuscript, to reflect the special situation in Germany and discussed the limitations.

3) Very importantly, German institutions have to be explained to the international readership: For example: “events like “Nur Mut - ChirurgIn werden” [9] and “Staatsexamen und Karriere” [10] by the german berufsverband” A non German reader will not understand what that means and what kind of event those are.

Answer: A table is now provided explaining these German institutions or campaigns and most German term are erased from the text, where possible.

4) You talk about lot of gender issues in surgery, the discussion of the literature as well as the discussion of the current guidelines for pregnant surgeons could be more thorough.

Answer: Thank you, we have added evidence for this point, however guidelines for pregnant surgeons or their application are quite different between countries and concerning Germany even between the different federal states. Giving an overview of the available legal restrictions or recommendations would go beyond the scope of this paper.

5) What about “#meetoo in medicine/surgery?

Answer: We appreciate the input, however we do not see where this issue ties in to this article about societal work. It raises another

discussion that is neither within the scope of this article, not the scope of the special issue in which it shall be published.
Regardless, we agree that sexual harassment is an important issue in medicine and particularly in surgery; however we strongly feel that the discussion needed for this would put the article out of focus and proportion, thus we chose to not address this issue.
6) There is a lot of international literature available on adverse events in pregnancy for surgical residents as well as publications on gender specific quality of treatement for female vs male surgeons.
Answer: The whole paragraph about surgery during pregnancy has been revised and literature has been added. However, limiting the discussion is that the mainly American literature is based on different working conditions and laws. There, many pregnant surgical residents do not change their working habits (because they don't have the option to do so) probably leading to more adverse effects. The aim of the OPIDS initiative is to find a balance to keep the pregnant surgeon working in surgery, but yet to meet maternity protection laws. The initiative does not aim at exploiting the pregnant surgeon and expose her to relevant hazards.
7) How can these problems be affected with more active societal work, expecially what can young surgeons do? You mentioned some, but overall the discussion should be more scientific. I would rephrase the section "The Personal side" in "Personal motivation" Regarding the section of personal motivation: one should consider if the names should be mentioned. My suggestion would be: male/female, age, training status (intern/resident/with training year). I would also wish to hear more about what the authors/persons mentioned have already achieved for them or others through the work in a surgical society.
Answer: Thank you for this comment. Concerning the personal achievements within the young surgeon committees the statements were revised as suggested.
Concerning the pseudonymization, we do not think this is necessary. We are proud to be associated with our work and opinion and feel that by withholding names the personal level of these statements would be lost to some extend. As such we chose to leave them in the manuscript for now.
If you would however see this as inappropriate we can always remove them as part of another revision.
In this context we also like to mention the following point: not every state in medical training or every position in the hospital hierarchy in Germany is similar to the ones existing in the US or UK. And then again we would have to choose whether we "translate" our position in the American or British system. This might not make the paper more international. It is however possible to add the amount of years in training regardless of the current position.
Thank you again for all the comments.

Reviewers' Comments to Revision

Reviewer 1: anonymous

Nov 27, 2018

**Reviewer Recommendation Term:**	Accept
**Overall Reviewer Manuscript Rating:**	50
**Custom Review Questions**	**Response**
Is the subject area appropriate for you?	4
Does the title clearly reflect the paper's content?	4
Does the abstract clearly reflect the paper's content?	4
Do the keywords clearly reflect the paper's content?	3
Does the introduction present the problem clearly?	4
Are the results/conclusions justified?	4
How comprehensive and up-to-date is the subject matter presented?	3
How adequate is the data presentation?	4
Are units and terminology used correctly?	4
Is the number of cases adequate?	N/A
Are the experimental methods/clinical studies adequate?	N/A
Is the length appropriate in relation to the content?	3

Does the reader get new insights from the article?	3
Please rate the practical significance.	2
Please rate the accuracy of methods.	N/A
Please rate the statistical evaluation and quality control.	N/A
Please rate the appropriateness of the figures and tables.	3
Please rate the appropriateness of the references.	3
Please evaluate the writing style and use of language.	3
Please judge the overall scientific quality of the manuscript.	3
Are you willing to review the revision of this manuscript?	Yes

Comments to Authors:
No further comments.

Reviewer 2: anonymous

Nov 19, 2018

**Reviewer Recommendation Term:**	Accept
**Overall Reviewer Manuscript Rating:**	N/A

**Custom Review Questions**	**Response**
Is the subject area appropriate for you?	4
Does the title clearly reflect the paper's content?	4
Does the abstract clearly reflect the paper's content?	4
Do the keywords clearly reflect the paper's content?	4
Does the introduction present the problem clearly?	4
Are the results/conclusions justified?	4
How comprehensive and up-to-date is the subject matter presented?	4
How adequate is the data presentation?	4
Are units and terminology used correctly?	4
Is the number of cases adequate?	N/A
Are the experimental methods/clinical studies adequate?	N/A
Is the length appropriate in relation to the content?	4
Does the reader get new insights from the article?	4
Please rate the practical significance.	3
Please rate the accuracy of methods.	N/A
Please rate the statistical evaluation and quality control.	N/A
Please rate the appropriateness of the figures and tables.	4
Please rate the appropriateness of the references.	4
Please evaluate the writing style and use of language.	4
Please judge the overall scientific quality of the manuscript.	3
Are you willing to review the revision of this manuscript?	No: no revision

Comments to Authors:
Good response to the reviewer.
